# Mucin-producing urothelial-type adenocarcinoma of the prostate with a gene mutation characteristic of intestinal adenocarcinoma: case report and literature review

**DOI:** 10.3389/fmed.2024.1494952

**Published:** 2025-01-20

**Authors:** Ao Yu, Hongbo Su, Peiling Yu, Siqi Cai, Shuaixian Mu, Jinhui Yu, Qianting Lu, Yuan Miao, Ailin Li

**Affiliations:** ^1^Department of Radiotherapy, Cancer Hospital of China Medical University, Liaoning Cancer Hospital and Institute, Cancer Hospital of Dalian University of Technology, Shenyang, Liaoning, China; ^2^School of Graduate, China Medical University, Shenyang, China; ^3^Department of Pathology, The First Affiliated Hospital and College of Basic Medical Sciences, China Medical University, Shenyang, Liaoning, China

**Keywords:** mucin-producing adenocarcinoma, urothelial-type adenocarcinoma, prostate, gene sequencing, radiotherapy

## Abstract

We report an elderly male with mucin-producing urothelial-type adenocarcinoma of the prostate (MPUAP) and oligometastatic lung involvement, initially diagnosed as benign prostatic hyperplasia and treated with transurethral plasma resection of the prostate (TURP). Postoperative pathology indicated mucinous adenocarcinoma, with immunohistochemistry positive for CK7, CK20, and CDX-2. Next-generation sequencing (NGS) identified genetic alterations similar to those found in intestinal adenocarcinoma. After ruling out gastrointestinal and bladder tumors, MPUAP was confirmed. Ablation therapy was performed for the lung metastasis, followed by radical prostate chemoradiotherapy. Post chemoradiotherapy, the patient received XELOX + Bevacizumab regmien but switched to capecitabine monotherapy due to adverse effects. At a 12-month follow-up post-radiotherapy, no prostate recurrence was observed, though previous lung nodule ablation suggested recurrence. By reviewing historical cases, we discussed the role and significance of radical resection and TURP in MPUAP. NGS is recommended for patients with MPUAP, and regarding chemotherapy, treatment options for colorectal cancer are worth considering.

## 1 Introduction

Mucin-producing urothelial-type adenocarcinoma of the prostate (MPUAP) represents an exceedingly rare malignancy of the prostate. Early clinical symptoms and imaging findings often deviate from the typical, thereby presenting a diagnostic challenge. Furthermore, there exists limited literature on MPUAP and no standardized treatment protocols. In this study, we present a case of MPUAP, conduct a review of pertinent literature, and consolidate clinical characteristics, pathological features, and treatment modalities associated with MPUAP. We aim to provide clinicians with comprehensive guidance for the diagnosis and treatment of this rare malignancy. This paper is the first to report that the results of next-generation sequencing (NGS) of MPUAP were consistent with the molecular characteristics of intestinal tumors, which not only guided the treatment plan for this case but also highlighted the potential significance of NGS in the diagnosis and management of this disease.

## 2 Case presentation

Patient, male, 77 years old, with a history of lower limb venous thrombosis. Since 2020, he has experienced progressively worsening urinary frequency, weak urination, and increased nocturia. In February 2023, an ultrasound examination suggested benign prostatic hyperplasia. Given the normal prostate specific antigen (PSA) levels (total PSA 1.430 ng/ml), he was diagnosed with benign prostatic hyperplasia at a local hospital and underwent transurethral resection of the prostate (TURP) to alleviate symptoms. However, postoperative pathology indicated malignancy, prompting his referral to our department.

**FIGURE 1:**
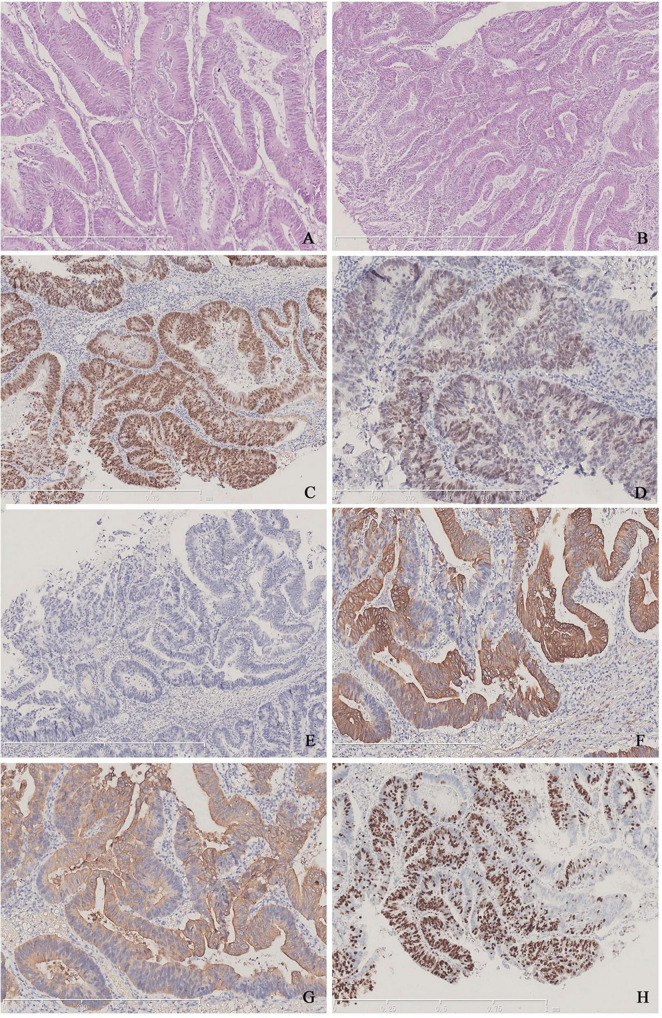
**(A,B)** Typical tall columnar tumor cells arranged closely and forming irregular glands of varying sizes, characteristic of colorectal villous-tubular adenoma. **(C)** Positive staining for CDX-2 in tumor cells. **(D)** Dim expression of SATB2 in the nuclei. **(E)** Lack of PSA expression. **(F)** Positive findings for CK7. **(G)** Positive findings for CK20. **(H)** The Ki-67 index is approximately 70%.

Firstly, we conducted tests for serum tumor markers and re-reviewed the pathological specimens. Serum tumor markers were as follows: carcinoembryonic antigen (CEA) 14.93 ng/ml, CA-199 40.59 U/ml, and total PSA 0.617 ng/ml. Pathologically, the morphology of the specimen was consistent with MPUAP, but tumors originating from the intestines or bladder needed to be excluded. The tumor displayed tall columnar cells forming irregular glandular structures, similar to colorectal villotubular adenoma, with occasional papillary formations. Additionally, urothelial adenomatous metaplasia and focal necrosis were observed. Immunohistochemistry was negative for PSA, NKX3.1, P63, and GATA3, but positive for CK7, CK20, β-catenin, CDX2, and SATB2 (Figure 1). To confirm the diagnosis, we performed cystoscopy and gastrointestinal endoscopy, both of which revealed no abnormalities, leading to a final diagnosis of MPUAP.

Next, we performed MRI and PET-CT to assess the tumor burden. MRI showed no enlarged lymph nodes, no signs of malignancy in the local prostate area post-surgery, and no abnormalities in the surrounding tissues. PET-CT revealed increased FDG metabolism in the residual prostate tissue, consistent with postoperative changes, and identified a 1.5 × 1.3 cm irregular fluorodeoxyglucose hypermetabolic nodule in the upper lobe of the right lung, suggesting metastasis (Supplementary Figure 1). We recommended a biopsy but the family declined due to the patient’s poor condition. Consequently, in March 2023, we performed radiofrequency ablation on the lung lesion. NGS analysis of MPUAP has been rarely reported in the existing literature. Given the morphological similarities between MPUAP and colorectal cancer, as well as CDX2 positivity (a hallmark of gastrointestinal tumors), our pathologists recommended NGS to further characterize the tumor at the genetic level. The NGS results were surprising, revealing multiple gene mutations, including PIK3CA, TP53, APC, KRAS, ARID1A, and RNF43, which are frequently observed in colorectal cancer (Table 1). Although a metastatic origin from the intestine was ruled out, the tumor’s morphology, immunohistochemical profile, and genetic sequencing results collectively led us to adopt a colorectal cancer chemotherapy regimen for this patient. Genetic testing further revealed a PD-L1 TPS of <1%, microsatellite stability (MSS), and a low tumor mutational burden (TMB); therefore, immunotherapy was not considered.

**TABLE 1:** Next generation sequencing analysis of the patient.

	Nucleotide alteration	Amino acid alteration	Exon	Abundance of alteration
PIK3CA	g.3:178936092 NM_006218.4 c.1634A > C	p.E545A	exon10	17.34%
KRAS	g.12:25380275 NM_004985.5 c.182_183delinsTG	p.Q61L	exon3	15.66%
TP53	g.17:7577570 NM_000546.6 c.711G > T	p.M237I	exon7	24.35%
APC	g.5:112175951 NM_00038.6 c.4666dup	p.T1556Nfs* 3	exon16	9.14%
ARID1A	g.1:27059260 NM_006015.6 c.1897C > T	p.Q633*	exon4	5.46%
RNF43	g.17:56435702 NM_017763.6 c.1433_1434del	p.S478Cfs* 26	exon9	20.80%
EPHA5	g.4:66230893 NM_001281766.3 c.2015G > C	p.R672P	exon11	19.06%
DNAH2	g.17:7683487 NM_020877.5 c.5735G > A	p.R1912H	exon37	27.19%
RBM10	g.X:47040980 NM_005676.5 c.1510G > C	p.A504P	exon14	16.76%
KMT2A	g.11:118372450 NM_001197104.2 c.6383C > A	p.P2128H	exon26	48.01%

* Indicates frameshift mutations at certain mutation sites.

Considering the patient’s lung metastasis, he was not suitable for radical prostatectomy. In April 2023, we initiated pelvic radiotherapy in conjunction with capecitabine chemotherapy. The clinical target volume (CTV) was delineated by the common, external, and internal iliac arteries, as well as the abdominal presacral and obturator lymphatic drainage areas. The planned target volume (PTV) dose was 46 Gy/23 F. The primary tumor CTV (CTVp) encompassed the prostatic bed, with the primary tumor PTV dose set at 76 Gy/38F (Supplementary Figure 2). Two weeks post radiotherapy, serum tumor markers were reassessed, revealing the following results: CEA 9.63 ng/ml, CA-199 35.5 U/ml, and total PSA < 0.006 ng/ml. Subsequently, the patient underwent one cycle of bevacizumab combined with XELOX chemotherapy. After one cycle of chemotherapy, the tumor markers further decreased: CEA 7.84 ng/ml, CA-199 31.93 U/ml, and total PSA < 0.006 ng/ml. Abdominal CT scans showed no progression in the pelvis, and chest CT scans showed a 2.6 × 1.4 cm nodule in the anterior segment of the right upper lobe, interpreted as an ablation response. Due to coagulation abnormalities and a history of lower limb venous thrombosis, bevacizumab was discontinued, and oxaliplatin was stopped due to an allergic reaction. The treatment regimen was adjusted to capecitabine monotherapy for 6 months.

Following radiotherapy, the patient was monitored through regular follow-up for 12 months. The most recent evaluation showed: CEA 7.38 ng/ml, CA-199 27.65 U/ml, and total PSA < 0.035 ng/ml, with no progression of the local prostate tumor. However, the nodule in the right upper lobe had increased to 2.7 × 1.7 mm, with lobulation, spiculation, and pleural traction, accompanied by mediastinal lymph node enlargement, indicating lung tumor recurrence. Due to the patient’s advanced age, the family declined further treatment. Therefore, the decision was made to continue best supportive care while maintaining regular follow-up.

## 3 Discussion

Research on MPUAP has primarily consisted of case reports. MPUAP is a rare tumor, making diagnosis challenging. Unlike prostate acinar adenocarcinoma, increased serum PSA levels are seldom observed in MPUAP. Differential diagnosis should initially rule out metastatic tumors originating from the intestines and bladder, as well as prostate adenocarcinoma. Histologically, MPUAP tumor cells typically exhibit high columnar or cubic shapes with varying degrees of atypia, arranged in tubular and cribriform structures. Tumor cells secrete abundant mucus, forming mucus lakes that separate the stroma. Our case also presents these characteristics. Immunohistochemically, our case showed positivity for CK7, CK20, and CDX2, consistent with prior MPUAP reports. CDX2, commonly expressed in gastrointestinal tumors, has also been identified in tumors with mucinous differentiation across various organs (1). While CDX2-positive staining is rare in prostatic cancer (2, 3), it was relatively common in previously reported MPUAP cases.

Given the rarity of MPUAP, it is essential to explore and investigate this tumor from more perspectives. Currently, clinical diagnosis relies primarily on conventional histopathological analysis. Integrating genetic mutation profiling into this process can provide valuable insights into the intrinsic biological characteristics of tumors. Tumor gene mutations are not entirely random; specific oncogenes often exhibit co-occurring mutations within the same tumor type (4). This observation suggests that tumors could be classified based on their genetic mutation profiles, which not only enhances diagnostic accuracy but also indicates that patients with similar genomic characteristics often share comparable clinical features and therapeutic responses (5). The significance of genetic mutations in MPUAP remains unclear. Among previously reported MPUAP cases, only two by Moe et al. provided genetic phenotypes, both showing concurrent mutations in FAT1 and HNF1A (6). Notably, our case is the first to report genetic alterations in MPUAP that resemble those observed in colorectal cancer. NGS analysis in our patient revealed mutations in genes such as PIK3CA, TP53, APC, KRAS, and RNF43, among others. These mutations are frequently seen in colorectal cancer (7), whereas KRAS, PIK3CA, and APC mutations are rarely observed in prostate cancer (8–12). This NGS finding not only supported our treatment decision but also innovatively emphasized the importance of incorporating comprehensive NGS analysis into the diagnostic workup for MPUAP patients, providing valuable guidance for future cases.

Aggressive treatment strategies can improve the survival rate of rare, high-grade incidental prostate cancer, with significantly lower other-cause mortality compared to patients who did not receive active treatment—this is associated with a poorer quality of life (13–15). A review of previous studies indicates that radical resection or TURP was performed in all cases with reported treatment details. Notably, none of the cases exhibited distant metastasis at the time of diagnosis. Given the potential poor prognosis associated with MPUAP, radical resection remains recommended when feasible. Curtis et al. and Fukiage et al. reported two cases with T2N0M0 staging who underwent radical resection and were followed up for 16 months and 4 years, respectively, without tumor recurrence or metastasis (16, 17). However, Camacho et al. reported a case with T3N0M0 staging who underwent radical resection without adjuvant therapy, resulting in local tumor recurrence after 15 months of follow-up (18). Therefore, adjuvant therapy after surgery is considered necessary for patients with more advanced tumor staging.

In earlier literature, eight cases of MPUAP patients underwent TURP. Among these, four cases underwent TURP initially due to a diagnosis of benignity. In the remaining four cases, tissue obtained through TURP allows for further diagnostic clarification, while also serving as a component of palliative or curative treatment strategies. Among eight cases, two patients underwent TURP alone (16, 19). Given that TURP cannot control tumor progression, multimodal therapy becomes imperative. Endocrine therapy is generally deemed ineffective. Ortiz-Rey et al. reported a case of a patient with a serum PSA elevation of 11.8 ng/ml, which stands as the only reported MPUAP case with elevated PSA. This patient underwent TURP combined with endocrine therapy but unfortunately died of the disease after 40 months of follow-up (20). Among the remaining five patients, one underwent chemotherapy, and four underwent radical radiotherapy to the prostate (two of whom received chemoradiotherapy (21, 22). Radical radiotherapy emerges as a dependable treatment option. Niu et al. and Guo et al. reported on two patients who underwent TURP combined with RT and were followed up for 12 and 30 months, respectively, without tumor recurrence or metastasis (23, 24).

We conducted a thorough analysis of MPUAP chemotherapy regimens cited in the literature. Given the morphological and immunohistochemical similarities between MPUAP and colorectal cancer, there has been an increasing trend in recent years toward reporting the use of colorectal cancer chemotherapy protocols for treating MPUAP (21, 22, 25, 26). One case reported promising outcomes: Solakhan et al. documented a RAS- and RAF-negative patient who underwent TURP followed by 76 Gy/38F radiotherapy in conjunction with hormone therapy. Subsequently, at 9 months post-surgery, multiple bone metastases, iliac lymph node metastases, and suspected pulmonary nodules were detected. The patient exhibited resistance to docetaxel-based prostate cancer regimens, androgen deprivation therapy, and gemcitabine-based bladder cancer regimens. Ultimately, treatment with a chemotherapy regimen designed for metastatic colon cancer, combined with panitumumab, achieved a positive response (22). In summary, while comparing the efficacy of different protocols based on a limited number of cases remains challenging, such efforts are meaningful and offer valuable insights for future research.

In our case, the patient initially underwent TURP following a diagnosis of benign prostatic hyperplasia. After confirming the diagnosis of MPUAP, PET-CT indicated pulmonary metastases, making the patient unsuitable for radical surgery. Consequently, we opted for definitive radiotherapy concurrent with capecitabine chemotherapy. Given the tumor’s morphological and immunohistochemical resemblance to colorectal cancer, along with highly concordant NGS results indicative of colorectal cancer characteristics, we confidently selected the first-line treatment regimen recommended by the NCCN rectal cancer guidelines for patients with unresectable primary tumors and isolated lung metastasis: 5FU-based chemotherapy combined with targeted therapy (XELOX + Bevacizumab) (27). The aim was to maximize the patient’s survival time while reducing the rates of local recurrence and distant metastasis. However, due to thrombus formation, oxaliplatin hypersensitivity and poor performance status, the treatment plan was ultimately adjusted to oral capecitabine monotherapy. At the 1-year follow-up after radiotherapy, no evidence of local prostate tumor recurrence was observed, but the previously ablated lung metastasis showed signs of recurrence. At this point, the patient’s family opted for best supportive care. This outcome highlights two key observations: first, the treatment regimen achieved excellent local control of the primary tumor; second, for metastatic lesions, surgical resection could be considered as a more definitive local approach when the initial conditions allow, and it is also necessary to adjust the second-line treatment regimen after recurrence.

Nonetheless, this study represents a single case, and generalizing these findings remains challenging. Further exploration and studies are needed to better understand and optimize treatment strategies for similar cases in the future.

## 4 Conclusion

Next-generation sequencing plays a crucial role in improving our understanding of the tumor’s intrinsic characteristics and classification. We report for the first time a case of MPUAP with genetic alterations resembling those of colorectal cancer. Radical resection remains the preferred treatment for MPUAP. In cases where radical surgery is not feasible, TURP can provide tissue for accurate pathological diagnosis, and subsequent definitive chemoradiotherapy represents the best alternative. For chemotherapy, adopting regimens used for colorectal cancer appears to be a promising approach. Unlike traditional reliance on pathological morphology and immunohistochemistry, the colorectal cancer-like genetic alterations observed in our case support the use of colorectal cancer chemotherapy regimens. Given the disease’s high invasiveness, follow-up examinations indicate significant efficacy of our treatment regimen in achieving local prostate control. For the ablated lung metastasis that later recurred, we recommend employing more definitive local treatment options at initial diagnosis and further exploring chemotherapy strategies.

Our understanding of this disease remains limited, underscoring the importance of ongoing data collection and case reporting to define its clinical behavior and establish optimal treatment strategies for this rare and aggressive disease.

## Data Availability

The original contributions presented in this study are included in this article/Supplementary material, further inquiries can be directed to the corresponding authors.
