# Supplementary material for: Mucin-producing urothelial-type adenocarcinoma of the prostate with a gene mutation characteristic of intestinal adenocarcinoma: case report and literature review
Source: Front Med (Lausanne). 2025 Jan 20;11:1494952. doi: 10.3389/fmed.2024.1494952 (PMC11789685; doi:10.3389/fmed.2024.1494952)
Supplement: Supplementary file 1 [file Image_1.pdf]

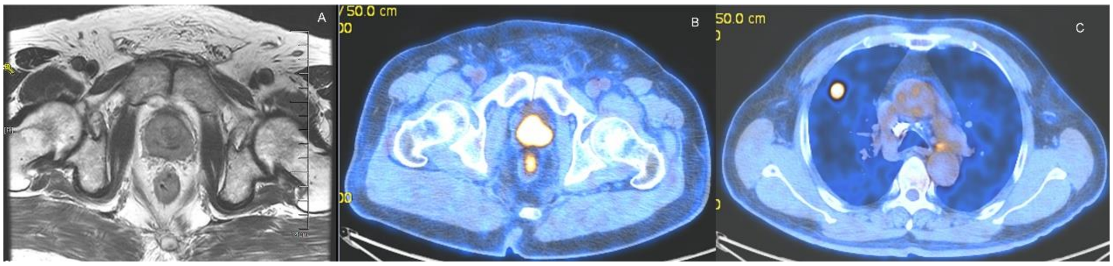

Supplement 1. (A) Pelvic enhanced magnetic resonance imaging suggested postoperative changes in the prostate. (B) After prostatic surgery, the residual FDG metabolism increased, consistent with postoperative changes. (C) A high FDG metabolic nodule in the upper lobe of the right lung was considered as a metastatic lesion.
