# Supplementary material for: Mucin-producing urothelial-type adenocarcinoma of the prostate with a gene mutation characteristic of intestinal adenocarcinoma: case report and literature review
Source: Front Med (Lausanne). 2025 Jan 20;11:1494952. doi: 10.3389/fmed.2024.1494952 (PMC11789685; doi:10.3389/fmed.2024.1494952)
Supplement: Supplementary file 2 [file Image_2.pdf]

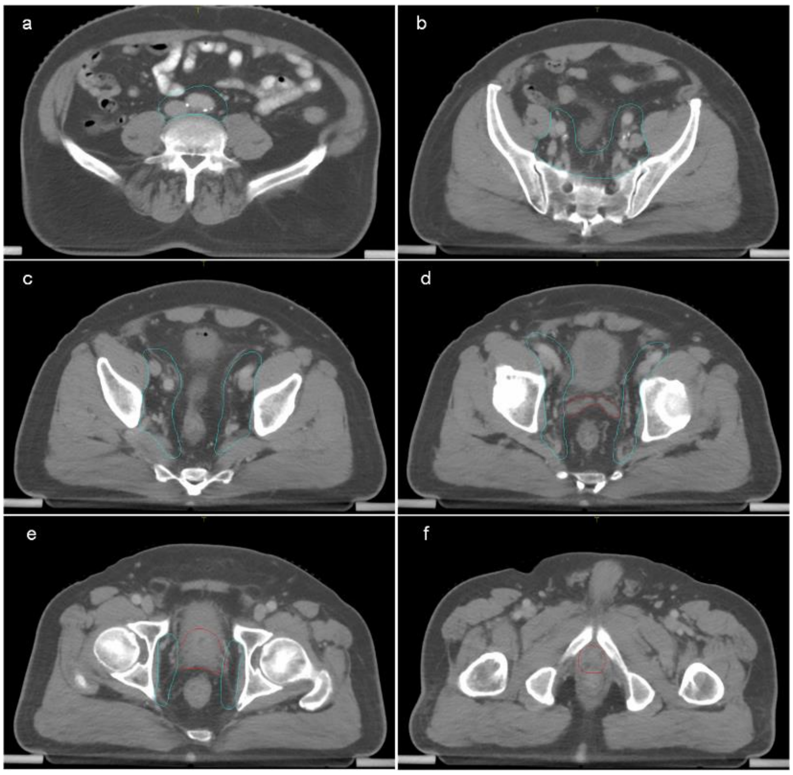

Supplement 2. Clinical target volume (CTV [blue]) and primary tumor clinical target volume (CTVp [red]) on the simulation computed tomography (CT). (A) CT image showing the upper border of CTV, indicating the abdominal presacral lymph node drainage area. (B) CT image showing the upper middle layer of CTV, illustrating the internal iliac, external iliac, and pelvic anterior sacral lymph node drainage areas. (C) CT image showing the middle layer of CTV, revealing the exposed piriformis muscle, as well as the internal and external iliac lymph node drainage areas. (D) CT image at the middle and lower layers of CTV and the upper bounds of CTVp, displaying the internal iliac, external iliac, and obturator lymph node drainage areas, as well as the seminal vesicle glands. (E) CT image at the lower border of CTV, indicating the obturator lymph node drainage area. CTVp depicts the middle layer of the prostate gland. (F) CT image at the lower border of CTVp, located 5 mm above the penile bulb.
